# Quantitative Analysis of Mutant Subclones in Chronic Myeloid Leukemia: Comparison of Different Methodological Approaches

**DOI:** 10.3390/ijms17050642

**Published:** 2016-04-29

**Authors:** Sandra Preuner, Agnes Barna, Florian Frommlet, Stefan Czurda, Byrgazov Konstantin, Mary Alikian, Katerina Machova Polakova, Tomasz Sacha, Johan Richter, Thomas Lion, Christian Gabriel

**Affiliations:** 1 Children’s Cancer Research Institute (CCRI), Zimmermannplatz 10, A-1090 Vienna, Austria; sandra.preuner@ccri.at (S.P.); stefan.czurda@ccri.at (S.C.); konstantin.byrgazov@ccri.at (B.K.); 2 Red Cross Transfusion Service for Upper Austria, A-4017 Linz, Austria; agnes.barna@o.roteskreuz.at (A.B.); christian.gabriel@o.roteskreuz.at (C.G.); 3 Department for Medical Statistics, Medical University of Vienna, A-1090 Vienna, Austria; florian.frommlet@meduniwien.ac.at; 4 Imperial Molecular Pathology Laboratory, Hammersmith Hospital, Imperial College Healthcare National Health Service (NHS) Trust, London W12 0HS, UK; m.alikian@imperial.ac.uk; 5 Institute of Hematology and Blood Transfusion, 128 20 Prague, Czech Republic; katerina.machova@lf1.cuni.cz; 6 Hematology Department, Jagiellonian University, 31-501 Krakow, Poland; sachatom@gmail.com; 7 Section for Hematology, Department of Medicine, University Hospital of Lund, 221 00 Lund, Sweden; johan.richter@med.lu.se; 8 Department of Pediatrics, Medical University Vienna, A-1090 Vienna, Austria

**Keywords:** *BCR-ABL1*, CML, quantitative analysis of mutant subclones, NGS, LD-PCR, pyrosequencing

## Abstract

Identification and quantitative monitoring of mutant *BCR-ABL1* subclones displaying resistance to tyrosine kinase inhibitors (TKIs) have become important tasks in patients with Ph-positive leukemias. Different technologies have been established for patient screening. Various next-generation sequencing (NGS) platforms facilitating sensitive detection and quantitative monitoring of mutations in the *ABL1*-kinase domain (KD) have been introduced recently, and are expected to become the preferred technology in the future. However, broad clinical implementation of NGS methods has been hampered by the limited accessibility at different centers and the current costs of analysis which may not be regarded as readily affordable for routine diagnostic monitoring. It is therefore of interest to determine whether NGS platforms can be adequately substituted by other methodological approaches. We have tested three different techniques including pyrosequencing, LD (ligation-dependent)-PCR and NGS in a series of peripheral blood specimens from chronic myeloid leukemia (CML) patients carrying single or multiple mutations in the *BCR-ABL1* KD. The proliferation kinetics of mutant subclones in serial specimens obtained during the course of TKI-treatment revealed similar profiles via all technical approaches, but individual specimens showed statistically significant differences between NGS and the other methods tested. The observations indicate that different approaches to detection and quantification of mutant subclones may be applicable for the monitoring of clonal kinetics, but careful calibration of each method is required for accurate size assessment of mutant subclones at individual time points.

## 1. Introduction

The clinical implementation of tyrosine kinase inhibitors (TKIs) over a decade ago has revolutionized treatment of chronic myeloid leukemia (CML) but the occurrence of resistance, most commonly attributable to point mutations in the tyrosine kinase domain (TKD) of the *BCR-ABL1* fusion gene, remains an important challenge [1,2,3,4,5]. Timely identification of mutations associated with TKI resistance is therefore of great relevance for the clinical management of CML patients, and specific recommendations for mutational screening are provided by the European Leukemia Net (ELN) and the National Comprehensive Cancer Network *(*NCCN*)* [6,7,8]. The currently recommended and most commonly used technique for the detection of mutations is bidirectional Sanger sequencing of PCR-amplified *BCR-ABL1* fragments encompassing the entire TKD [6,7,9,10]. Due to its detection limit in the range of 10%–20%, this technique only facilitates assessment of relatively large mutant subclones, and permits only rough estimation of their size [6,7,9,10]. Since the detection of a mutation does not necessarily imply imminent onset of resistant disease [11,12,13], monitoring of the proliferation kinetics of mutant subclones during TKI treatment can provide more relevant clinical information [14,15]. In order to permit early detection of mutations, and to assess the biological behavior of mutant subclones during therapy, a number of sensitive methods facilitating quantitative monitoring have been developed with the aim to establish a basis for timely and rational clinical decisions [15,16,17,18,19]. Although serial measurement of *BCR-ABL1* levels by reverse-transcription real-time quantitative PCR remains the mainstay of patient surveillance during treatment [7], we have recently reported that the expansion of mutant subclones can be observed even prior to detection of rising fusion gene transcripts [15]. These observations underline the potential of sensitive and quantitative mutational analyses to provide early information on impending resistant disease. A variety of methodological approaches to detection and quantitative monitoring of mutant *BCR-ABL1* subclones have been published over the past few years, including allele-specific real-time PCR [20], pyrosequencing [12,18], ligation-dependent PCR techniques (LD-PCR; L-PCR) [19,21], methods based on various other principles [22,23,24,25,26], and, most recently, next-generation sequencing (NGS)-based methods exploiting different technical platforms [14,16,17,27]. The indicated methods are superior to Sanger sequencing in terms of sensitivity and ability to determine the size of mutant subclones. However, they display major differences with respect to relevant parameters, including the detection limit (ranging from 0.05% to 5%), the accuracy of quantitative analysis (if reported), and the clinical applicability with regard to technical prerequisites and overall costs. The NGS platforms offer many advantages over other approaches to detection and quantitative monitoring of mutant *BCR-ABL1* subclones [14,16,17,27], and will likely become the leading technology for this and other clinically relevant applications. However, the present costs of analysis and the limited accessibility of appropriate diagnostic services hamper their clinical implementation at many centers, thus emphasizing the current need for alternative techniques. We have therefore selected two well-established methods displaying detection limits in a range similar to NGS (≥1%) including pyrosequencing [18] and LD-PCR [19], and compared their performance in quantitative analysis of mutant *BCR-ABL1* subclones to next generation sequencing on the MiSeq (Illumina, San Diego, CA, USA) or GS junior/FLX+ (Roche, Basel, Switzerland) platforms. We have analyzed individual and serial peripheral blood specimens from CML patients harboring single or multiple point mutations in the *BCR-ABL1* TKD, and demonstrate that the methods tested show similar results with regard to the assessment of subclone kinetics. However, the differences observed between measurements of clonal size at individual time points highlight the need for appropriate calibration of any technical approach employed.

## 2. Results

**Figure 1:**
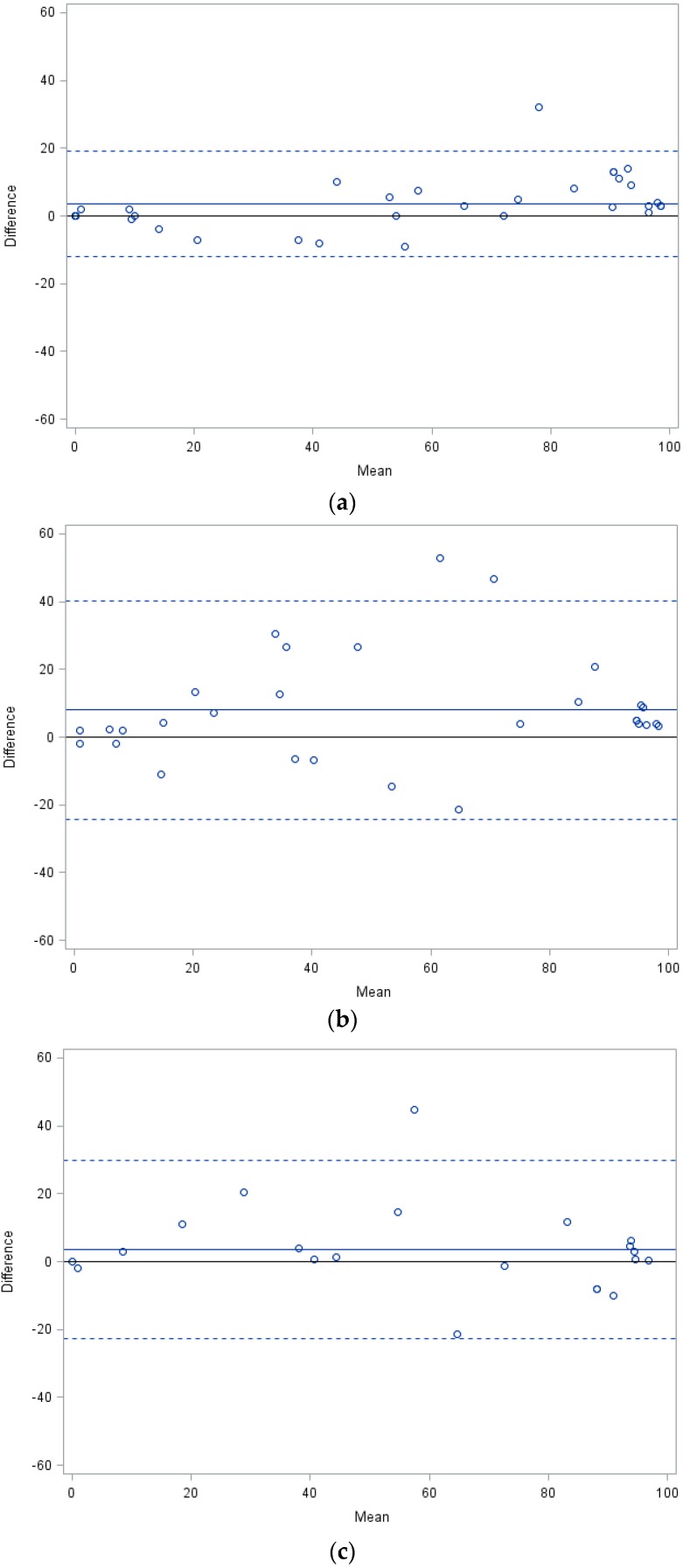

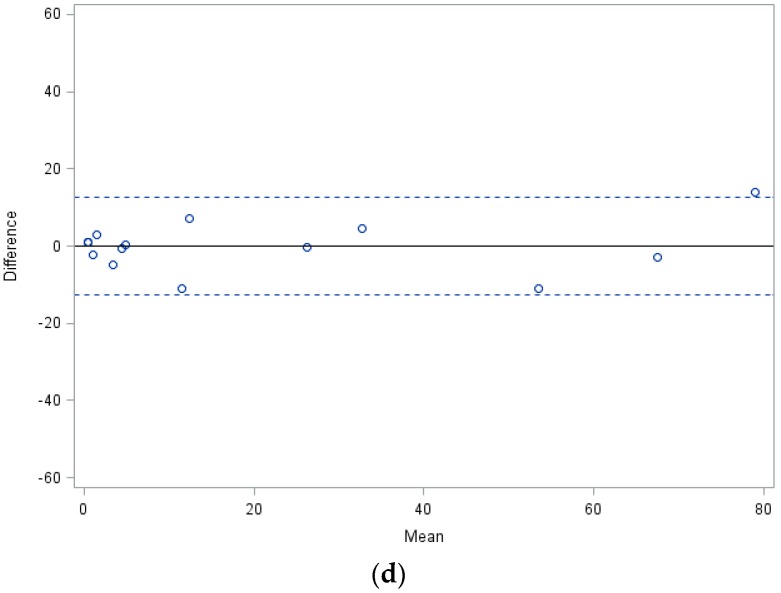
Comparison of quantitative mutant subclone assessment by different methods. The Bland-Altmann plots displayed show the distribution of mean ∆ values (∆*M*) with the respective 95% confidence intervals (dashed lines) for the pairwise comparison of (**a**) LD-PCR *versus* pyrosequencing-∆1; (**b**) LD-PCR *versus* NGS (MiSeq platform)-∆2; (**c**) pyrosequencing *versus* NGS (MiSeq platform)-∆3; (**d**) LD-PCR *versus* NGS (GS Junior platform). The number of data points varies between different pairwise comparative analyses because a proportion of cDNA specimens were not amenable to testing by all technical approaches. In some instances, individual points represent identical results of different measurements.

Of 105 cDNA samples derived from peripheral blood of CML patients carrying point mutations in the *BCR-ABL1* TKD, 46 specimens including both individual and serial samples passed the initial quality control, and could therefore be subjected to quantitative comparison of subclone size assessment by different technical approaches. This limitation indicated that storage of clinical specimens under suboptimal conditions can be a major impediment for ensuing molecular analyses requiring high quality of RNA/cDNA. The parameters of quantitative analysis by the LD-PCR method including accuracy, reproducibility, and limit of detection had been previously established and characterized at our center, and provided a basis for the measurements performed. The analyses were based on the investigation of specimens obtained from different clinical centers and comprised two independent data sets, one including the pairwise and comprehensive comparison between LD-PCR, pyrosequencing and NGS on the MiSeq (Illumina) platform, and the other the comparison of LD-PCR with NGS on the GS Junior (Roche) platform. The absolute quantitative values obtained by the different methods are displayed in Table 1 and Table 2. The specimens analyzed within both data sets displayed nine different mutations including p.M244V, p.G250E, p.Q252H (c.756 G>T), p.Y253H, p.E255K, p.V299L (c.895 G>T), p.T315I, p.M351T, and p.F359V. Individual patients revealed up to three mutant subclones but none of them were present in compound constellations, as determined by NGS. Results of the first dataset are illustrated by Bland-Altmann plots showing the differences in measurement against the average of all pairwise-compared methods tested (Figure 1a–c). The comparison of differences between LD-PCR and pyrosequencing (∆1) revealed virtually all data points within the 95% confidence interval (CI), with a single exception, and indicated a trend towards slightly higher values determined by the LD-PCR method (Figure 1a). Comparison of LD-PCR and NGS on the MiSeq platform (∆2) showed only two values outside the 95% CI, but displayed greater divergence between paired sample analyses than LD-PCR and pyrosequencing (Figure 1b). A more pronounced disparity was observed in the comparison of differences between pyrosequencing and NGS on the MiSeq platform (∆3) but all data points, with a single exception, were still within the 95% CI (Figure 1c). Analysis of the second dataset based on a different series of cDNA specimens, permitting only comparison of LD-PCR with NGS on the GS junior platform, revealed the most concordant results with a narrow range of the 95% CI (Figure 1d).

**Figure 2:**
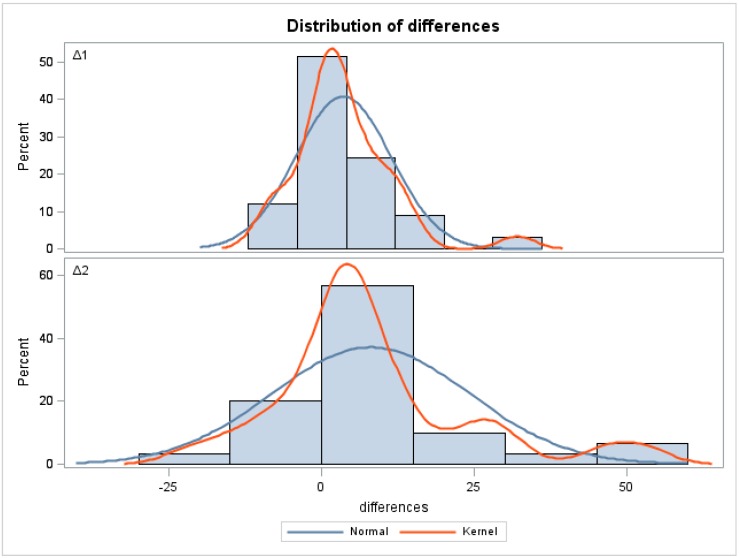
Distribution of ∆*M* values. Histograms and corresponding density estimators for the differences between methods are displayed. The **upper** panel represents ∆1, the difference between LD-PCR and pyrosequencing, and the **bottom** panel reflects ∆2, the difference between LD-PCR and NGS on the Miseq platform. The values determined by LD-PCR and pyrosequencing showed greater similarity than those obtained by LD-PCR and Miseq-NGS. The normal density (blue line) clearly shows greater variance for ∆2 in comparison to ∆1.

The distribution of differences including ∆1 (LD-PCR *versus* pyrosequencing) and ∆2 (LD-PCR *versus* NGS-MiSeq platform) is illustrated by histograms and corresponding density estimates (Figure 2). The central tendency of ∆2 is slightly larger than that of ∆1, but the difference is not statistically significant (Table 3). The much wider spread observed in ∆2 compared to ∆1 indicates a greater similarity between results obtained by LD-PCR and pyrosequencing than between LD-PCR and NGS on the MiSeq platform, which is highly significant according to a folded *F* Test (*p* = 0.0001).

The smallest differences between quantitative measurements were seen in the comparison of LD-PCR with NGS on the GS Junior platform using a small set of samples (*n*: 15, mean ∆ value: −0.074, Standard Deviation (SD): 6.3). However, due to the different set of cDNA specimens employed for this test series, statistical analysis correlating the data to the pairwise comparisons performed within the other test series was not possible (see Table 2 for quantitative results).

**Figure 3:**
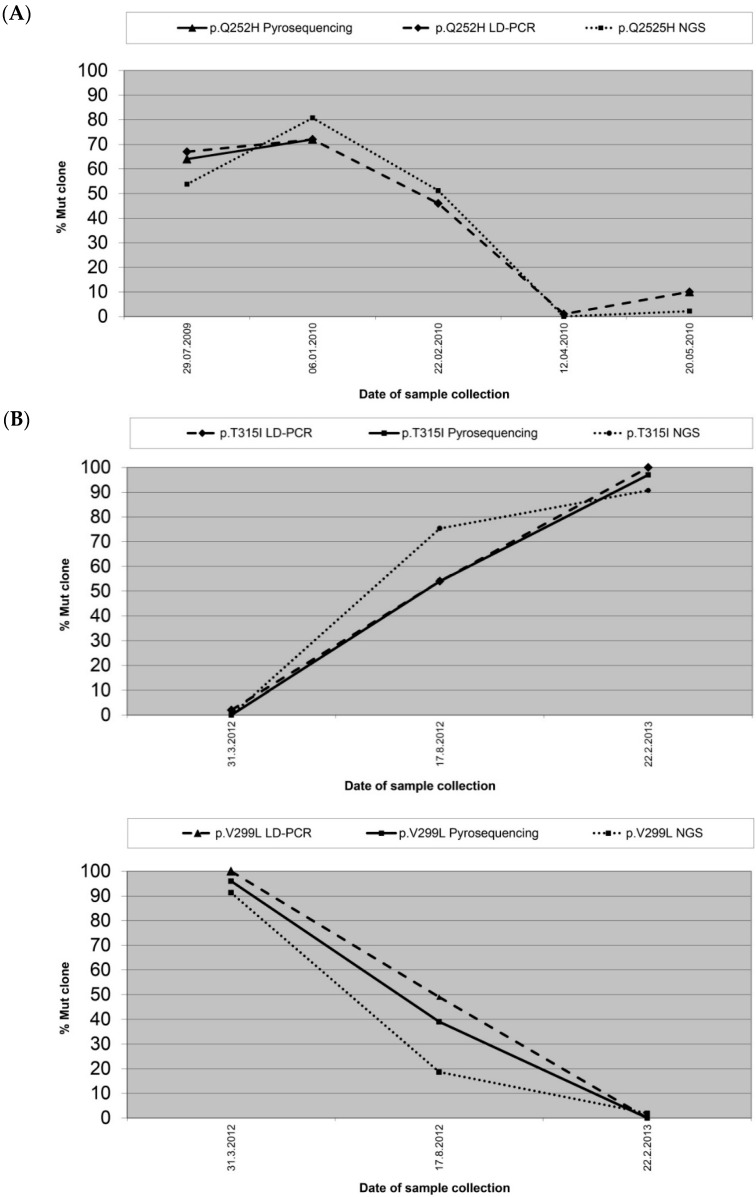
Comparison of mutant subclone monitoring by different methods. Serial specimens from CML patients carrying (**A**) a single mutation, p.Q252H (c.756G>T) or (**B**) two independent mutations, p.T315I (**upper** panel) and p.V299L (c.895 G>T) (**lower** panel), are displayed showing the monitoring by LD-PCR (dashed line), pyrosequencing (solid line), and NGS on the FLX+ Roche platform (dotted line). The concordant kinetics, as determined by all technical approaches employed, are highlighted particularly by the largely overlapping curves of analyses by LD-PCR and pyrosequencing.

In addition to the comparison of quantitative measurements in individual specimens, the availability of serial samples from nine patients permitted the assessment of proliferation kinetics of mutant subclones by different techniques. Two examples comparing quantitative monitoring of mutant subclones in consecutive specimens by LD-PCR, pyrosequencing and NGS are displayed in Figure 3. The concordant kinetics documents the similarity of results generated by the three methods with regard to the dynamics of subclone evolution.

## 3. Discussion

Detection of mutations in the *BCR-ABL1* TKD has become an important parameter for the management of patients with Ph-positive leukemias [15,16,28,29], and monitoring of the size of mutant subclones indicative of their sensitivity or resistance to treatment can be expected to take on more clinical importance in the foreseeable future. Although a variety of techniques greatly expanding the information provided by Sanger sequencing were introduced [14,16,17,18,19,20,21,27], studies on direct comparison of different quantitative approaches have been lacking. Clinically relevant detection levels for mutant subclones have not been determined to date, indicating that the monitoring of kinetics may be a more informative parameter. The accuracy of quantitative measurement of mutant subclones as well as the sensitivity of detection could be of importance for adequate patient surveillance. Improvement of the detection limit offered by Sanger sequencing in conjunction with the ability to accurately quantify mutant subclones has therefore been the main challenge for all new technical approaches. Various NGS platforms meeting this requirement have been recently introduced, and their important advantages include the ability to screen the entire TKD for the presence of single and multiple mutations, to identify polyclonal and compound constellations in the presence of more than one mutation, and to determine the relative sizes of all mutations present at individual time points [14,16,17,27]. Some NGS techniques were shown to facilitate coverage of the complete *BCR-ABL1* TKD and even adjacent sequences of potential clinical relevance in a single long read, thus permitting more economic identification of compound mutations [14,27]. However, the practical detection limit of NGS analyses seems to be in the range of 1% [14,16,17,27], when searching for unknown mutations, due to the need to reliably exclude mutational artifacts by implementing appropriate bioinformatic algorithms. We have therefore selected methods providing a similar detection limit, including LD-PCR and pyrosequencing [18,19], for the comparison of performance with regard to size assessment of mutant subclones. Other technical approaches such as RT-qPCR [20,25] or digital PCR (Bio-Rad, Hercules, CA, USA) certainly represent additional alternative options, provided that the mutations to be monitored are known. The number of specimens available for comparative analyses was restricted by the stringent quality criteria and the required amount of material. This limitation resulted in variable sample numbers for the pairwise comparison of the methods tested, thus requiring appropriate statistical analysis for the evaluation of all measurements. Comparison of the data generated by LD-PCR, pyrosequencing and NGS on the Miseq platform revealed concordant kinetics, but individual values were similar between the former two methodologies, and showed significant differences to the values determined by NGS. Since the LD-PCR technique was precisely calibrated for accurate quantitative analysis [19], the differences observed should raise the awareness of the need to carefully calibrate NGS-based approaches for size assessment of mutant subclones.

The highest level of concordance was observed in the comparison of LD-PCR and NGS on the GS junior platform, but the smaller number of samples analyzed and the use of a different set of cDNA specimens for this test series must be considered. The different NGS approaches involved could not be systematically compared due to the limited amount of available specimens. Nevertheless, data generated on different NGS platforms were obtained in two instances. The anecdotal observations revealed differences between individual NGS platforms similar to those detected between the other methods tested, possibly suggesting a good level of concordance but no uniformity of results provided by different NGS approaches.

## 4. Experimental Section

### 4.1. Material

#### Patient Material and Ethical Statement

Archived cDNA specimens from CML patients previously identified to display mutations in the BCR-ABL1 TKD were obtained from different clinical centers including the Institute of Hematology and Blood Transfusion, Prague, Czech Republic; the Hematology Department, Jagiellonian University, Krakow, Poland, the Section for Hematology, Department of Medicine, University Hospital of Lund, Sweden and the Hammersmith Hospital, London, UK (Ethical approval, REC reference: 06/Q0406/47). In the present study, a total of 105 specimens were tested by different technical approaches to mutational analysis. The specimens analyzed included exclusively archived residual cDNA from patients who had previously undergone molecular mutational analysis with informed consent at the centers involved.

Initial control of the amount and quality of cDNA was performed by photometric measurement and PCR amplification of the *ABL1* gene [30]. Forty six samples revealing *ABL1* copy numbers ≥1 × 10^4^ per reaction were deemed eligible for quantitative analysis of mutant subclones.

### 4.2. Methods

#### Quantitative Analysis of Mutant *BCR-ABL1* Subclones

Eligible cDNA samples were investigated using different technical approaches to quantitative mutational analysis including LD-PCR, pyrosequencing and NGS [14,17,18,19,31]. The principle of LD-PCR relies on competitive hybridization of wildtype (WT) or mutation-specific oligonucleotide probes followed by PCR amplification and assessment of the PCR products by fragment length analysis on a sequencing machine. Technical details of LD-PCR have been described previously [19]. Pyrosequencing is based on sequence analysis by nucleotide chain synthesis, thus also permitting quantitative detection of multi-allelic mutations, which is not possible by the LD-PCR technique. Technical details of the pyrosequencing method have been described earlier [18]. Both methods facilitate size assessment of point-mutated subclones with a limit of detection in the range of 1%–5%. However, prior knowledge of the mutation to be analyzed is required, thus rendering these techniques inappropriate for screening purposes. By contrast, NGS permits both screening for unknown mutations with a detection limit of 1%, and quantitative analysis of individual or multiple mutations in a single reaction [17,31]. Recently established NGS technologies enable also the detection and quantification of multiple mutations within the same DNA molecule (compound mutations) by providing long reads covering the entire sequence of interest. Technical details of the long-range NGS approach employed were recently published [27]. The experimental design of the present study was based primarily on the LD-PCR method, for which all validation parameters of quantitative analysis are well documented [19]. Pairwise comparison of LD-PCR to pyrosequencing and NGS on two different platforms was performed on the basis of individual and serial clinical specimens from CML patients carrying mutant *BCR-ABL1* subclones. Table 4 shows a comparison of important parameters for all three technologies.

### 4.3. Statistical Analysis

Bland-Altman plots including mean values and 95% confidence intervals (CI) for differences between methods were used to determine the pairwise agreement between LD-PCR *versus* pyrosequencing (∆1), LD-PCR *versus* NGS on the Miseq platform (∆2), pyrosequencing *versus* NGS on the Miseq platform (∆3), and LD-PCR *versus* NGS on the GS Junior platform based on a different set of samples. Different statistical tests were performed to compare the disagreement of measurements with a focus on ∆1 and ∆2. A folded *F*-Test (SAS PROC TTEST) was performed to test the equality of variance between ∆1 and ∆2. Furthermore, a repeated-measures test ANOVA (SAS PROC MIXED) was employed to assess the equality of mean values between ∆1 and ∆2. Histograms with kernel densities are provided to illustrate the results (Figure 2).

## 5. Conclusions

The findings presented indicate that different methods facilitating sensitive detection of mutant subclones and quantitative assessment of their size, such as LD-PCR or pyrosequencing, can be regarded as eligible for clinical monitoring in terms of mutant subclone kinetics. However, the differences between the methods tested with regard to quantitative measurement of subclone size at individual time points highlight the need for appropriate calibration. Although NGS techniques will likely become the methods of choice for this application due to the important advantages outlined above, alternative techniques will be required as long as the costs of analysis and accessibility of appropriate diagnostic services prevents broad clinical application of this technology. However, the limitations in terms of the comparability of results generated by different methods must be taken into consideration. Depending on the local expertise and availability of laboratory equipment, different methods may permit adequate surveillance of *BCR-ABL1* TKD mutations relevant in the context of TKI resistance as a basis for optimal management of patients with Ph-positive leukemias.

## Figures and Tables

**Table 1:** Single and serial peripheral blood specimens from 19 CML patients displaying up to three different mutant subclones were subjected to quantitative analysis by LD-PCR, pyrosequencing and/or NGS on the MiSeq platform (Illumina). Serially derived specimens from individual patients are indicated by increasing numbers after the hyphen. The *BCR-ABL1* transcript levels in individual specimens are indicated according to the International Scale (IS). The sizes of mutant subclones determined by the technical approaches tested are displayed as a percentage of the *BCR-ABL1* positive cells. n.a., not analyzed; n.d., not detected; pos, detected but not quantified.

Sample Number	BCR-ABL1 % (IS)	Mutation	LD-PCR (% Mut)	Pyroseq (% Mut)	NGS MiSeq (% Mut)
1-1	0.670	p.E255K	97	84	92
1-2	1.700	p.E255K	100	86	96
2-1	9.834	p.E255K	82	pos	n.a.
2-2	0.667	p.E255K	97	84	92
2-3	2.338	p.E255K	97	86	n.a.
3-1	1.670	p.E255K	41	pos	28
3-2	4.180	p.E255K	49	pos	22
3-3	5.800	p.E255K	61	pos	35
3-1	1.670	p.Y253F	27	pos	20
3-2	4.180	p.Y253F	27	pos	14
3-3	5.800	p.Y253F	7	pos	5
3-1	1.670	p.T315I	6	pos	8
3-2	4.180	p.T315I	9	pos	20
3-3	5.800	p.T315I	3	pos	n.a.
4-1	20.015	p.T315I	92	89	n.a.
4-2	15.692	p.T315I	56	50	n.a.
4-3	13.075	p.T315I	62	54	n.a.
5-1	18.550	p.V299L	100	96	91
5-2	1.380	p.V299L	49	39	19
5-3	20.670	p.V299L	0	0	2
5-1	18.550	p.T315I	2	0	0
5-2	1.380	p.T315I	54	54	75
5-3	20.670	p.T315I	100	97	91
6-1	41.355	p.F359V	10	8	n.a.
6-2	29.897	p.F359V	51	60	n.a.
6-3	10.786	p.F359V	12	16	n.a.
6-4	11.302	p.F359V	0	0	n.a.
7-1	0.080	p.F359V	n.a.	n.a.	67
7-2	0.050	p.F359V	88	80	35
7-3	0.120	p.F359V	17	24	13
8	6.280	p.G250E	n.d.	pos	0
9	20.060	p.T315I	98	89	77
9	20.060	p.G250E	n.a.	n.a.	33
10-1	0.108	p.Q252H	67	64	n.a.
10-2	0.200	p.Q252H	72	72	n.a.
10-3	0.079	p.Q252H	46	n.a.	61
10-4	0.016	p.Q252H	1	n.a.	n.a.
10-5	0.020	p.Q252H	10	10	n.a.
11	1.990	p.M244V	90	pos	80
12	2.670	p.T315I	97	96	93
13	35.570	p.E255K	94	62	47
14	8.640	p.M244V	77	72	73
14	8.640	p.Y253H	9	10	7
15-1	18.218	p.T315I	0	0	n.a.
15-2	21.540	p.T315I	0	0	n.a.
15-3	15.313	p.T315I	37	45	44
16	18.500	p.Y253H	98	95	94
17	14.370	p.T315I	34	41	40
18	0.040	p.Y253H	n.a.	40	36
19	0.160	p.T315I	100	97	97

**Table 2:** Displayed are serial specimens from three CML patients, each carrying two or more different mutant subclones, which were analyzed by LD-PCR and NGS on the GS junior platform (Roche). n.a., not analyzed.

Sample Number	BCR-ABL1 % (IS)	Mutation	LD-PCR (% Mut)	NGS GS Junior (% Mut)
1-1	7.200	p.M351T	86	72
1-2	0.180	p.M351T	n.a.	26
1-1	7.200	p.G250E	5	5
1-2	0.180	p.G250E	1	0
2-1	5.900	p.T315I	26	26
2-2	15.000	p.T315I	66	69
2-1	5.900	p.M351T	1	6
2-2	15.000	p.M351T	1	0
2-1	5.900	p.Y253H	3	0
2-2	15.000	p.Y253H	6	17
3-1	0.140	p.M351T	48	59
3-2	2.300	p.M351T	16	9
3-3	8.400	p.M351T	1	0
3-1	0.140	p.F359V	35	30
3-2	2.300	p.F359V	4	5
3-3	8.400	p.F359V	0	2

**Table 3:** Comparison of quantitative mutant subclone assessment using different methods. Overview of results obtained by pairwise comparison of measurements by LD-PCR, pyrosequencing, and NGS on the Miseq platform (based on quantitative values displayed in Table 1). The number of samples amenable to comparison by different methods varies because a proportion of cDNA specimens were not large enough for analysis by all technical approaches. The mean values are not significantly different according to statistical analyses performed by the two sample *T*-Test (*p* = 0.17) or the more refined ANOVA test based on repeated measurements (*p* = 0.11), indicating that none of the methods has an obvious bias towards larger or smaller values. However, the SD of ∆2 (16.1) in comparison to ∆1 (7.8) is significantly higher according to the folded *F*-Test (*p* < 0.0001), indicating the most pronounced differences in test results obtained by LD-PCR and NGS (Miseq).

Specifications	LD-PCR/Pyrosequencing	LD-PCR/NGS	Pyrosequencing/NGS
Number of samples compared (N)	33	30	21
Difference of mean values (∆*M*)	3.5	8	3.6
Standard deviation (SD)	7.8	16.1	13.1

**Table 4:** Performance features of the methods compared.

Performance Features	LD-PCR	Pyrosequencing	NGS
Quantitative analysis of mutant subclones	yes	yes	yes
Prior knowledge of mutation required	yes	yes	no
Detection of multiple mutations in one reaction	no	yes	yes
Detection limit for mutant subclones	1%–5%	5%	1%
Accuracy of quantification	±5%	n.a.	n.a.

n.a., not available.
